# Evaluation of deepfake detection using YOLO with local binary pattern histogram

**DOI:** 10.7717/peerj-cs.1086

**Published:** 2022-09-13

**Authors:** Štěpán Hubálovský, Pavel Trojovský, Nebojsa Bacanin, Venkatachalam K

**Affiliations:** 1 Department of Applied Cybernetics, University of Hradec Králové, Hradec Králové, Czech Republic; 2 Department of Mathematics, Faculty of Science, University of Hradec Králové, Hradec Kralove, Czech Republic; 3 Department of Computing, Singidunum University, Belgrade, Serbia

**Keywords:** Deepfake, YOLO, LBPH, FaceForencies++, Celeb-DF, Celeb DF-Face Forensics++

## Abstract

Recently, deepfake technology has become a popularly used technique for swapping faces in images or videos that create forged data to mislead society. Detecting the originality of the video is a critical process due to the negative pattern of the image. In the detection of forged images or videos, various image processing techniques were implemented. Existing methods are ineffective in detecting new threats or false images. This article has proposed You Only Look Once–Local Binary Pattern Histogram (YOLO-LBPH) to detect fake videos. YOLO is used to detect the face in an image or a frame of a video. The spatial features are extracted from the face image using a EfficientNet-B5 method. Spatial feature extractions are fed as input in the Local Binary Pattern Histogram to extract temporal features. The proposed YOLO-LBPH is implemented using the large scale deepfake forensics (DF) dataset known as CelebDF-FaceForensics++(c23), which is a combination of FaceForensics++(c23) and Celeb-DF. As a result, the precision score is 86.88% in the CelebDF-FaceForensics++(c23) dataset, 88.9% in the DFFD dataset, 91.35% in the CASIA-WebFace data. Similarly, the recall is 92.45% in the Celeb-DF-Face Forensics ++(c23) dataset, 93.76% in the DFFD dataset, and 94.35% in the CASIA-Web Face dataset.

## Introduction

Artificial intelligence techniques and deep learning algorithms facilitate the creation of fake videos and images in a realistic situation. The open source software, such as DeepFaceLab and FakeApp, was most commonly used to create fake images that are then publicly shared between social networking platforms. This false morphing creates serious problems in society (Zhang et al., 2016). The term “deepfake” is defined as changing the face in a photo from original to fake. Misusing images manipulates opinions of people and also affects important issues such as election results, corporate survival, *etc*. (Tan & Le, 2019). YOLO face detector is frequently used to detect the face using the frame detection technique.

Fake face detection is considered a the classification problem which divides the morphed face from the original videos. Recently, there have been numerous deepfake detection algorithms that are presented using deep learning techniques (Deng et al., 2019). Some of the methods for detecting fake faces in videos and images are (a) visual incongruity, (b) artifacts in frame detection, and (c) temporal incongruity based on deep convolutional and recurrent neural networks. Due to wide usage of social medias, there is standard requirement for fake face detection technology. There exist many face detection strategies, and it is necessary to improve existing algorithms to identify fake faces with high accuracy. As a result, it was implied that there exists a need to develop a method that requires more intelligent technology for deepfake detection.

YOLO (Ismail et al., 2021) is considered as a regression rather than a complex pipeline in the Convolutional Neural Network. The main advantage of the technology is that it sees the full image during training and testing of the input image. YOLO has superior performance in detecting target. The contextual data encodes data frame implicitly. It encodes both data classes and appearance. In addition, it recognizes object detection as a single regression problem. Here, the object passes as pixels straight from the image to coordinate with the bounding box. Another advantage of YOLO is that the optimization of full image detection is done directly. Fake face manipulation in celebrity images and videos creates great social problems. This deepfake must be detected before being distributed on large social networks.

The computer vision application uses the *local binary pattern histogram* (LBPH) for describing visually and classifying the images/videos. The detection performance is improved by using a histogram gradient by data vector. Also, some additional parameters used in the detection are radius, neighbors, grid *x*-horizontal, grid *y*-vertical vectors. Then the image is finally trained. The LBPH creates an intermediate image that is better than the real image to highlight facial features. The sliding window concept helps to highlight the facial characters in the LBPH images. Finally, the histograms are extracted with the region based on grids (*X*, *Y*) and concatenated the results of all histograms. So, the main advantage is that more local features represented in the image/video. Finally, images occur with robustness on transformations of monotonic grayscale. Our research provides an improved detection technique using a hybridized YOLO face detector with a Local Binary Pattern Histogram. This proposed technique helps to detect the fake faces in fast and efficient manner. The main contributions of this work are as follows.

1. Deepfake video detection is carried out using YOLO with local binary pattern detection with histogram gradients. So that, the detection is done fast and most easily.
2. Training the network on the real world image and understanding the generalized object representation by analyzing the local features using binary pattern
3. YOLO–LBPH architecture is similar to a Fully Convolutional Neural Network. The input is processed as n*n and the output is m*m grids. The difference is that each grid two bounding boxes with class probabilities are used. Finally, the histograms are calculated and concatenated.
4. YOLO–LBPH makes a smaller number of errors in the background compared to the fast recurrent neural network.
5. Objects are recognized as general and histogram representation by YOLO-LBPH. It performs well in the new domains by analyzing the features on a local and general basis.

The organization of this article is carried out in the following arrangement: The “Literature survey” explores information related to existing techniques and their drawbacks. The “Proposed YOLO-LBPH methodology” reveals in detail the proposed working principle and ideology. The “Result and discussions” evaluates the results of the proposed technique implemented. The “Conclusion” discusses the conclusions of the research and its outcome. It also provides certain implications for the future scope of the work.

## Literature Survey

The digital media is increased with fake videos or forged videos to finev tune the unexpected outcomes in the society (Zhang et al., 2016). This article proposes the extraction of distillation manually with augmentation of frames. Finally, forged image is classified using the CNN. Here, it disables the overfitting issue, but the main drawback of the technique is that CNN is a pipeline model, which consumes more time in the network.The training of the data is important step in the CNN. The attention algorithm is used by the authors (Kwon, Yoon & Park, 2020) in the feature map. The manipulated face region is extracted from fake images. Moreover, it takes more time to compute. In article (Maheswaran et al., 2017) spatial technique was adapted in the shallow learning technique. This research identify the fake faces by combining both the spectrum and the spatial image. Identify the forgery section with up-sampling artifacts so that transferability is improved.

Deepfake detection using the multi-attention technique was considered the fine-grained classification algorithm (Maheswaran et al., 2020). Here the gradients not considered in the image. Local features were collected efficiently, but they did not calculate all frames and gradients. This problem motivated the researcher to choose a local binary pattern with histogram computation. The research carried out in (Wang et al., 2020) had implemented the image manipulation method. It was identified by facial expression and also localized the forgery in the image. However manipulation works like photoshop and accuracy is result is not acquired. The main disadvantage of the technique was that it did not produce an optimal outcome in all images. The article had used light-weight extraction for the fake face detection in the input dataset. Further, the subspace learning technique was utilized to analyze the face image to filter the significant feature. Unfortunately, these techniques do not support dynamic models. In (Li et al., 2020), the problem of overfitting in fake face detection was tackled using deep learning models. On other side it consumes more time by concentrating on overfitting.

Color-based analysis was presented in the research article (Wang, Thome & Cord, 2017). The image pixels are corrected using the edge region analyzing techniques. The unwanted pixel values was eliminated finally. The transformer algorithm in (Deng et al., 2019) was used to remove the unrelated pixel values.unrelated values of the pixels are difficult to estimate. Their moving process was carried out by a CNN convolutional transformer. More preferred technique like CNN was expected with high performance. Furthermore, a vision transformer (Vit) was used with the attention technique. Efficient Net B7 (Kang et al., 2017) was used as the backbone for the detection of fake faces. Non-deep learning techniques are widely used in this manipulation frame detection. Furthermore, well-known classifiers such as SVM (Yang, Li & Lyu, 2019) were used to detect the region generated by splicing in the face image. The classification using multi-layer perceptron (Zhang et al., 2019) detects fake videos where landmarking was suggested. In article (Chen et al., 2021; Das et al., 2021) the face identification was made through safe adversarial with friend concepts.

## Proposed YOLO-LBPH Methodology

**Figure 1:**
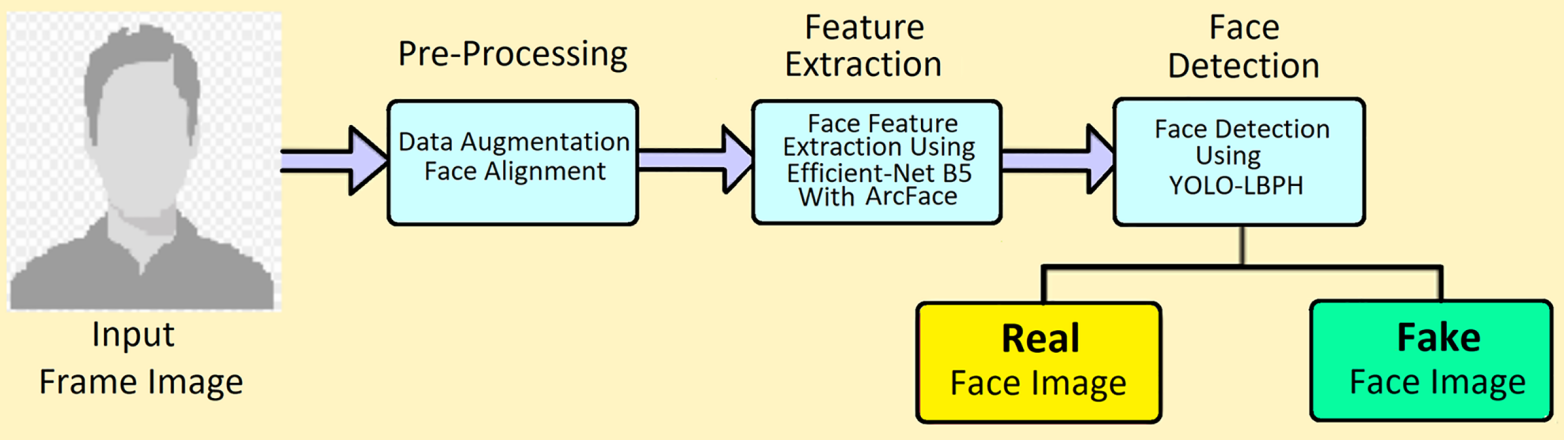
Architecture of deepfake video detection.

This digital era requires strong fake faces detection technology to monitor the forgery in short period. YOLO is the fastest fake face detection (Zhang et al., 2016) and the local binary pattern with a histogram would help increase the accuracy of fake manipulation in the original image. In addition, the binary patterns are used to analyse the frames in deep, and YOLO helps in fast training and data testing. In this section, the architecture of deepfake video detection is shown in Fig. 1.

In the pre-processing phase, the DF video input is divided into frames and detection is performed from the frames. First frames are cleared from external noises. This preprocessing step contains three main components; data augmentation, face detection, and face alignment. The main advantage of this proposed method is that using the circular neighborhood concept YOLO with LBPH works efficiently and fast. Yolo is most popularly used algorithm by concept of you only know once in identifying the face. It can detect faces with various fronts and sides of faces. Real-time detection is possible using this proposed algorithm.

### Pre-processing

**Figure 2:**
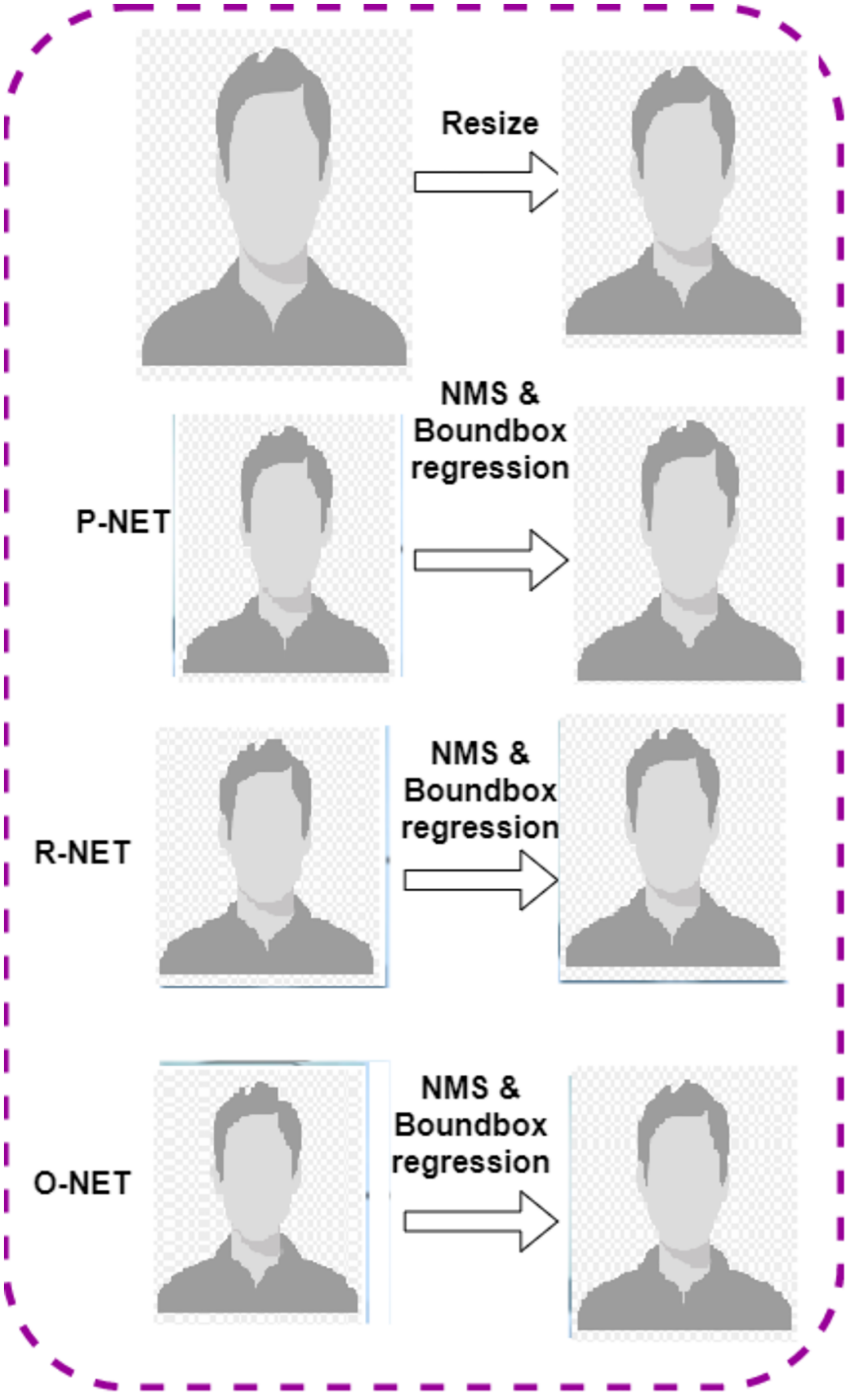
Workflow of the MTCNN model.

The pre-processing of fake videos is divided into frames using Multi-Task Cascaded Convolutional Neural Networks (MTCNN) (Zhang et al., 2016). The fake video detects the face image from the video frames using the MTCNN model. To enhance the image quality and the accurate detection of the fake image, the alignment of the face image is done. Here, faces are extracted from the video in 224 × 224 RGB format. After detecting the face image in the video, the MTCNN model is used to extract the face. This will lead to a reduction in the amount of misrelated information in the dataset. The MTCNN model is used for detecting the human face, which includes three stages; P-Net, R-Net, and O-Net. Figure 2 shows the workflow of the MTCNN model. The first figure is resized, and P-NET, R-NET, O-NET bound box regression is computed in sequential order.

The use of the MTCNN model reduces the computation time. The cascade structure of the MTCNN model has the following stages:

- **Stage 1:** P-Net detects the different regions of the face using a shallow convolutional network.
- **Stage 2:** R-Net is used to remove the non-face regions from the detected image.
- **Stage 3:** O-Net is used to produce fine output using a complex network architecture.

#### Data augmentation

In the training stage, the augmentation process is given below.

Zooming Augmentation is done by adding some new pixels to the input image; it will zoom the image randomly. Horizontal Flipping works by flipping a zoomed image using a Boolean value.

#### Face alignment

Fake face images are detected from the video by detecting the face alignment. It is used to target the various head positions of the person in the video frames.

### Face feature extraction

The feature extraction is considered as an essential step in extracting the facial features. To handle the larger size of videos, face detection is a challenging task in the aspect of quick access and accuracy. In this work, EfficientNet-B5 (Tan & Le, 2019) is used to extract facial features. To obtain a better and more robust face feature extraction, EfficientNet-B5 with additive loss of angular margin (also known as ArcFace) is used (Deng et al., 2019). The classification loss function (softmax loss) is defined as follows.

(1) $${L_1} = - \displaystyle{1 \over M}\sum\limits_{i = 1}^M {\log \displaystyle{{{e^{W_{{y_i}}^T{x_i} + {b_{{y_i}}}}}} \over {\sum\limits_{j = 1}^n {{e^{W_j^T{x_i} + {b_j}}}} }}} ,$$

where ${x_i} \in {{\mathbb R}^d}$ is the deep feature of the ${i^{{\kern 1pt} th}}$ sample that belongs to the $y_i^{{\kern 1pt} th}$ class (usually *d* is set to 512). ${W_j} \in {{\mathbb R}^d}$ is the ${j^{{\kern 1pt} th}}$ column of the weight $W \in {{\mathbb R}^{d \times n}}$ and ${b_j}$ is the bias term. The batch size and the class number are *M* and *n*, respectively. ArcFace is based on the concept of classification cross-entropy loss. The main aim of ArcFace is to reduce the intra-class features and enhance the inter-class features of the face image. In the training process, the steps needed to implement ArcFace are provided below:

In Algorithm 1, the bias value ${b_j} = 0$, then *logit* is transformed by $W_j^T{x_i} = \left\| {{W_j}} \right\|\left\| {{x_i}} \right\|  \cos {\theta _j}$, where ${\theta _j}$ is the angle between the weight ${W_j}$ and the feature ${x_i}$, where ||⋅|| is the usual vector norm ${\ell ^2}$. Furthermore, we fixed $||{W_j}|| = 1$ and the norm ${\ell ^2}$ of the feature ${x_i}$ we rescaled the value as *s* (the normalization step on the features and the weights makes the predictions depend only on the angle between the features and the weights). The learned embedding features are distributed on a hypersphere with radius *s*.

**Algorithm 1.** Pseudo-code of ArcFace.

**Input:** The face image features *img*, the parameter *p*, the number of classes *n*, and the ground truth *gndt*.
**Step 1:** In each class feature ${x_i}$ of the image *img*, with normalized weight *W*, produces $\cos {\theta _j}\ \left( {logit} \right)$.
**Step 2:** Evaluate arccos ${\theta _{{y_i}}}$ and the angle between the features ${x_i}$ and the weight ${W_{{y_i}}}$ of the ground truth.
**Step 3:** Add angular margin penalty *m* with angle ${\theta _{{y_i}}}$ of the ground truth value.
**Step 4:** Calculate $cos\left( {{\theta _{{y_i}}} + m} \right) \times logit$, where *logit* represents each class as the cosine of the angle between ground truth of hypersphere and feature, angular margin *m* (additional), the scale of feature from a small value to final logit *s*, this *logit* value process undergoes to implement cross-entropy loss.

(2) $${L_2} = - \displaystyle{1 \over M}\sum\limits_{i = 1}^M {\log } \displaystyle{{{e^{s\cos {\theta _{{y_i}}}}}} \over {{e^{s\cos {\theta _{{y_i}}}}} + \sum\limits_{j = 1,{\kern 1pt} j \ne {y_i}}^n {{e^{s\cos {\theta _j}}}} }}$$

In Step 3, the angular margin penalty *m* is added with the ground truth value. *m* is equal to the geodesic distance margin penalty in the normalized hypersphere, and the final loss is represented as

(3) $${L_3} = - \displaystyle{1 \over M}\sum\limits_{i = 1}^M {\log } \displaystyle{{{e^{s\cos ({\theta _{{y_i}}} + m)}}} \over {{e^{s\cos ({\theta _{{y_i}}} + m)}} + \sum\limits_{j = 1,{\kern 1pt} j \ne {y_i}}^n {{e^{s\cos {\theta _j}}}} }}$$

### Proposed face detection using YOLO-LBPH

**Figure 3:**
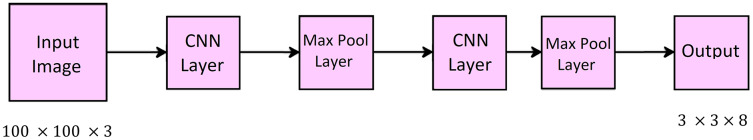
Framework of YOLO.

The input video image file is layered as frame by frame; whole frame of the video image is used to extract the features. it is considered as a tedious process. Therefore, the features are extracted only from the face region using a YOLO-LBPH face detector. You Only Look Once (YOLO) with Linear Binary Pattern Histogram (LBPH) is a face detector that detects faces from the frames of the video image. The main advantage of using hybrid technique of YOLO-LBPH is a faster detection of real-time object, and it also recognizes the image with less computational time complexity. The various frames of the videos helps to detects the head posture and angle diffrences in each frame for fake image identification. Read the input video image and divide the image into *img*1 × *img*1. Here, *img*1 is the grid size and is selected in random basis. Each cell of the grid represents multiple bounding boxes. This boxes used to predict certain aspects, such as class probabilities, an object, and confidence scores. The Non-Maximal Suppression (NMS) method is used for predicting the boxes. finally the overlapping boxes are remove. The every object is detected by NMS at once and false object is removed. Here, confidence score for each boundary box is predicted. This score is represented as probability of each class and detects only one object in a box. The boundary boxes are created by clustering the ground truth boxes from its dataset. Figure 3 shows the framework of YOLO for detecting the object.

The YOLO works based on Convolution Neural Network principle. It consists of a total of 24 convolutional layers followed by two fully connected layers. The first 20 convolutional layers are followed by an average pooling layer with fully connected layer, and it is a pre-trained data set of images with a resolution of 224 × 224 × 3. In addition to that, the training process of detecting the object needs the last four convolutional layers followed by two fully connected layers. The object detection is made comfortable with increasing resolution of the images in the dataset to 448 × 448. In the last layer, it predicts the probability of class and bounding boxes, and linear activation function is executed here, and the remaining layers implement the ReLU activation function.

LBPH is used to recognize facial images in the database. the binary operator is used to Extract facial features from image, it recognizes the image with less computational time complexity. Algorithm 2 describes the LBPH.

**Algorithm 2.** Pseudo-code of LBPH.

**Input:** An image of a face.
**Output:** LBP pixel values.
**Step 1:** Split the image into *n* ⋅ *m* matrix of subregions with *m* rows and *n* columns (thus, we have this image in grid form *n* × *m*).
**Step 2:** Extracting histogram values from each subregion $re{g_j}$, for $j = 1,2, \ldots ,n \cdot m$, of the face image using
(4) $$hist{o_{i,j}} = \sum\limits_{x,y} {Imge\ \left\{ {{f_{imge}}\left( {x,y} \right) = i} \right\}\;Imge\left\{ {\left( {x,y} \right) \in re{g_j}} \right\}} ,$$
with $i = 0,1, \ldots ,n - 1$ and $j = 0,1, \ldots ,m - 1$, where *m* is the total number of subregions and *n* is the total number of class labels created by the LBP operator.
**Step 3:** Apply the LBP operator in every subregion, and it is applied in the window size of 8 × 8 using the
(5) $$LBP\left( {i,j} \right) = \sum\limits_{t = 0}^{pix - 1} {{2^t}\;s\ \left( {im{g_t} - im{g_c}} \right)} ,$$
where $im{g_c}$ is the value of intensity of the center pixel and $im{g_t}$ is the value of intensity of a neighbor pixel of the center pixel, *s* is the following binary function
(6) $$s(x) = \left\{ {\matrix{ {1,\;x \ge 0;} \hfill \cr {0,\;x < 0.} \hfill \cr } } \right.$$
The idea of the function *s* is as follows. If the value of the intensity of a neighbor pixel value is greater than or equal to the value of the intensity of the center pixel, then the value of the function *s* is equal to 1; otherwise, it is equal to 0.
**Step 4:** Select the median pixel value as a threshold value and compare it with the neighborhood pixel value of the size of the image window 8 × 8.
**Step 5:** Combines all neighbor pixel values to form an 8 bit binary number and converts it into a decimal number (in ranges from 0 to 255), which is called the LBP pixel value.

In Algorithm 2, LBPH is the fusion of Local Binary Patterns (LBP) technique with the Histograms of Oriented Gradients (HOG) descriptor. It is a simple and powerful method to extract features and label pixels in the face image. LBPH is easy and time saving methodology.

## Result and Discussions

### Data collection

This proposed YOLO-LBPH is implemented by using the deepfake forensics (DF) dataset Celeb-DF-FaceForencics++(c23) which is a combination of Face Forencies++(c23) and Celeb-DF. For training the dataset, it randomly splits the dataset into a training sub-dataset and a validation sub-dataset. Pixels in the image are normalized to the range (−1, 1). The Face Forencies++ dataset and the Celeb-DF dataset are collected. The Celeb-DF dataset was originally divided into 5,299/712 to train the data and 340/178 to test the data as fake and real videos. Additionally, data from DFFD and CASIA-WebFace are collected.

### Performance metric measures

The evaluation of algorithm is done using the metrics like measuring mean square error and accuracy. To evaluate the performance of the proposed algorithm, it is compared with other existing approaches such as YOLO EfficientNet-B0 + [Bi-LSTM], YOLO + [XceptionNet] + [Bi-LSTM], YOLO + [XceptionNet] + [LSTM] (Ismail et al., 2021; Masi et al., 2020; Rossler et al., 2019; Güera & Delp, 2018). To evaluate the performance of the metric measures, such as the accuracy, sensitivity, specificity, and error rate of the Root Mean Square Error (RMSE), Signal-to-Noise-Ratio (SNR), Peak Signal-to-Noise-Ratio (PSNR), and Mean Absolute Error (MAE) are used. One of the evaluation metrics, the Area Under the Receiver Operating Characteristic (AUROC) curve, is used to evaluate the real and fake image using the proposed YOLO-LBPH.

(7) $$TPR = \displaystyle{{TP} \over {TP + FN}},$$

(8) $$FPR = \displaystyle{{FP} \over {FP + TN}}.$$

### Accuracy

(9) $$Aaccuracy = \displaystyle{{TP + TN} \over {TP + TN + FP + FN}} \cdot 100,$$

(10) $$Recall = \displaystyle{{TP} \over {TP + FN}},$$

(11) $$Precision = \displaystyle{{TP} \over {TP + FP}},$$

(12) $$F - Measure = 2{\mkern 1mu} \displaystyle{{Precision \cdot Recall} \over {Precision + Recall}},$$

The error rate is given by

(13) $$PSNR = 20\ {\log _{10}}\left( {\displaystyle{{{{255}^2}} \over {MAE}}} \right).$$

(14) $$MAE = \displaystyle{1 \over {MN}}\sum\limits_{i = 1}^M {\sum\limits_{j = 1}^N {\left| {X\left( {i,j} \right) - Y\left( {i,j} \right)} \right|} } ,$$

(15) $$RMSE = \sqrt {\displaystyle{1 \over N}\sum\limits_{i = 1}^N {{{\left( {{X_i} - \widehat {{X_i}}} \right)}^2}} } ,$$

(16) $$SNR(db) = 20\log \left( {\displaystyle{{{V_{RMS(Signal)}}} \over {{V_{RMS(Noise)}}}}} \right).$$

Table 1 shows the precision of various algorithms with the proposed work. The publicly available datasets are implemented such as FaceForencies++, Celeb-DF, DFFD, CASIA-Web Face. The accuracy rate of the proposed work YOLO-LBPH was 98.12% in the CASIA-Web Face image dataset. The next position in terms of the accuracy rate is 97.56% for the DFFD dataset.

**Table 1:** Performance comparison of the proposed YOLO-LBPH with other YOLO algorithms on different datasets in terms of accuracy.

Methods	Datasets			
	FaceForencies++ (c23)	Celeb-DF	DFFD	CASIA-WebFace
YOLO EfficientNet-B0 + [Bi-LSTM]	85.5	74.12	86.63	87.25
YOLO + [XceptionNet] + [Bi-LSTM]	86.11	76.11	89.45	85.52
YOLO + [XceptionNet] + [LSTM]	87.15	79.21	88.78	90.75
Proposed YOLO-LBPH	95.92	96.67	97.56	98.12

Table 2 shows the error detection rate for the proposed work for various dataset.

**Table 2:** Performance comparison of the proposed YOLO-LBPH with other YOLO algorithms on different datasets in terms of Error Detection Rate.

Methods	Datasets			
	FaceForencies++ (c23)	Celeb-DF	DFFD	CASIA-WebFace
YOLO EfficientNet-B0 + [Bi-LSTM]	14.73	28.81	14.34	13.67
YOLO + [XceptionNet] + [Bi-LSTM]	14.56	25.12	12.85	13.88
YOLO + [XceptionNet] + [LSTM]	12.13	15.34	14.67	11.24
Proposed YOLO-LBPH	8.12	10.15	8.14	6.16

**Figure 4:**
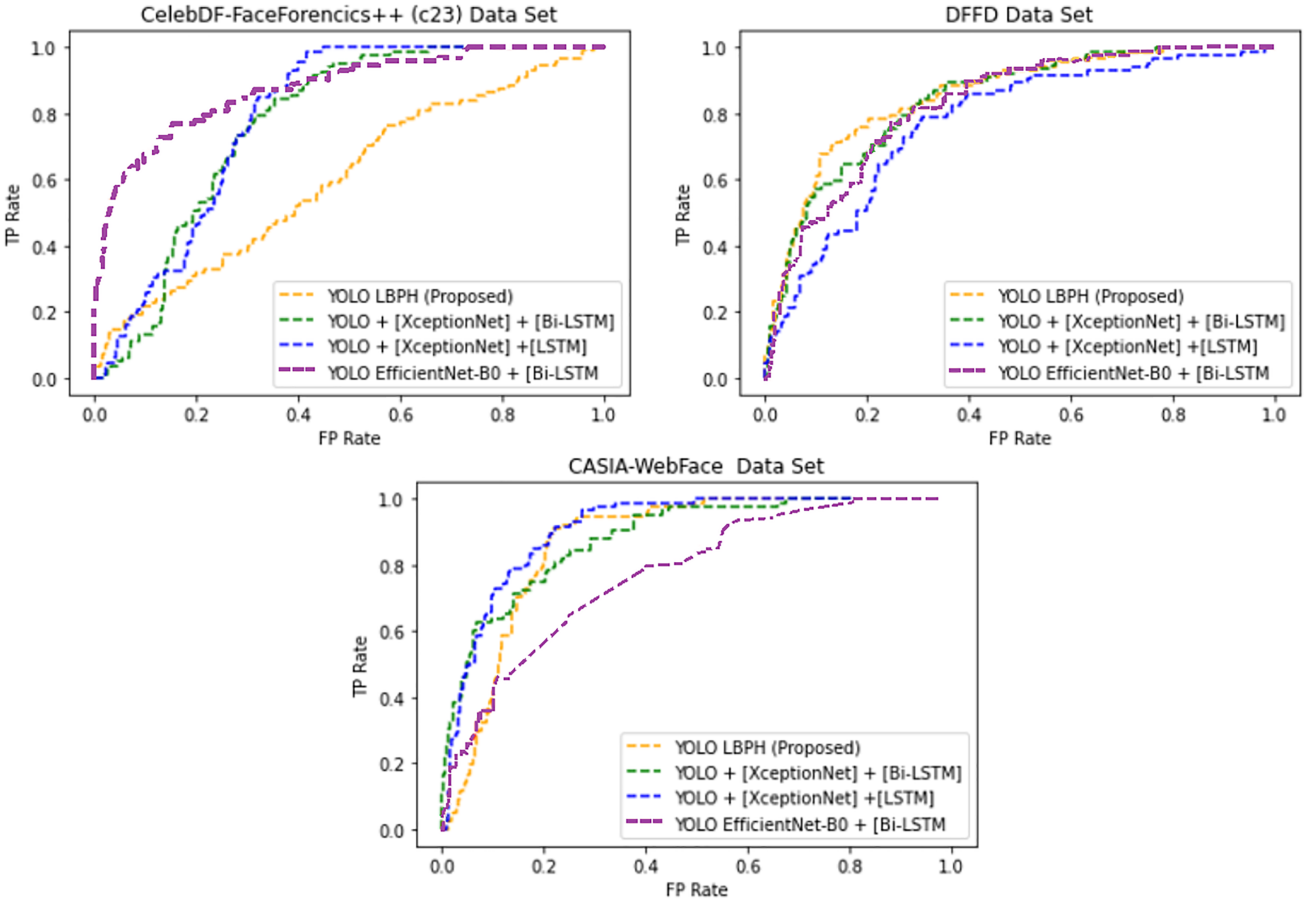
The Area Under the Receiver Operating Characteristic curve (AUROC).

Table 2 shows the mentioned algorithms error rate in different datasets. The proposed YOLO-LBPH had a minimum error rate equal to 6.16 for the CASIA-Web Face dataset. Fig. 4 shows the AUROC curve for the deepfake forensics (DF) datasets Celeb-DF-FaceForencics++(c23), DFFD and CASIA-WebFace, YOLO EfficientNet-B0 + [Bi-LSTM] has produced a better result.

**Figure 5:**
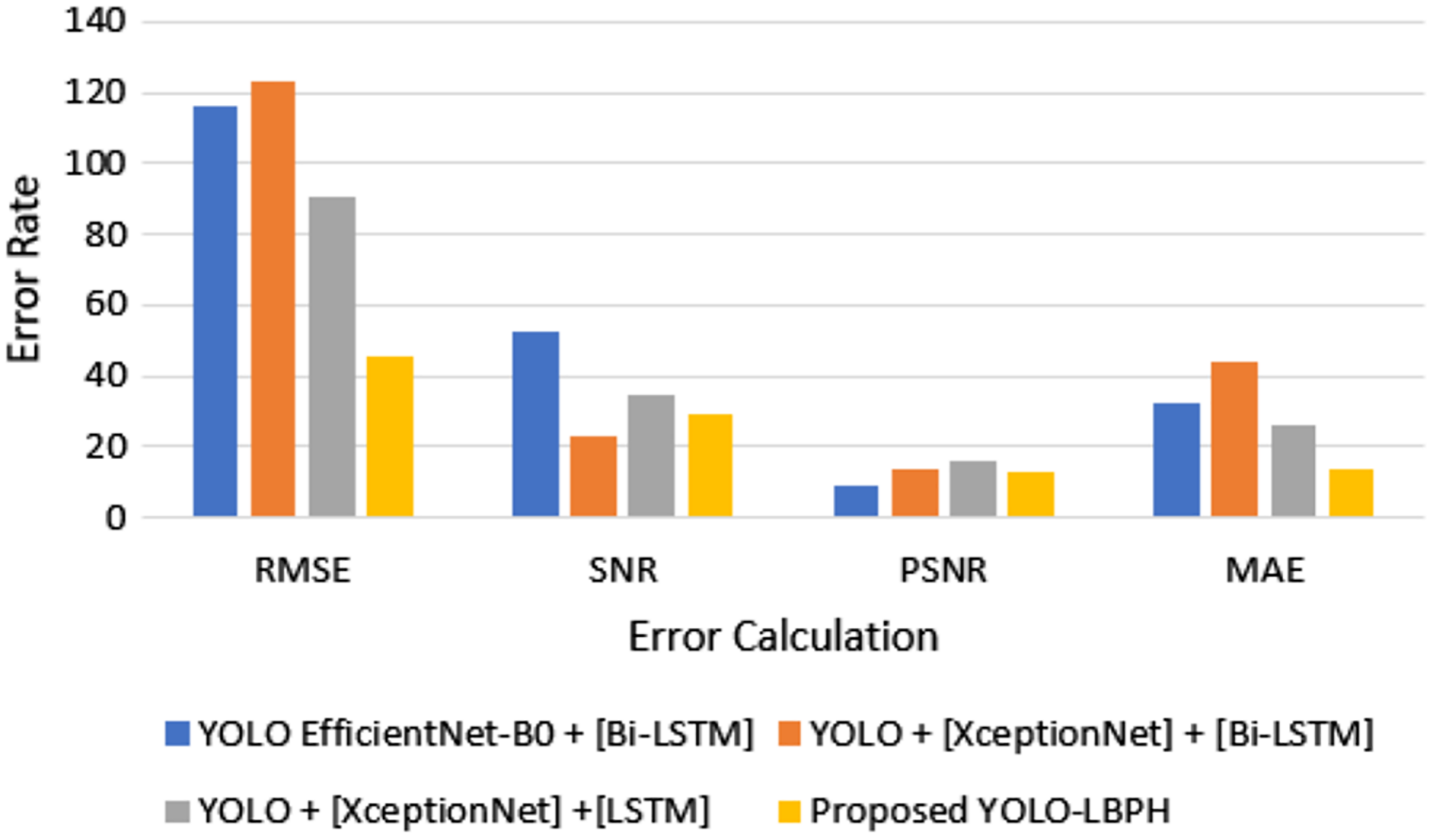
Error rate in accuracy.

Figure 4 shows that for the deepfake forensics (DF) dataset Celeb-DF-FaceForencics++(c23), the proposed work YOLO-LBPH has produced a better result compared to the other existing algorithms. In the DFFD dataset, YOLO + [XceptionNet] + [LSTM] has produced a better result in the CASIA-Web Face dataset. Figure 5 shows the error rate value calculated based on its accuracy by using Eqs. (13)–(16).

**Figure 6:**
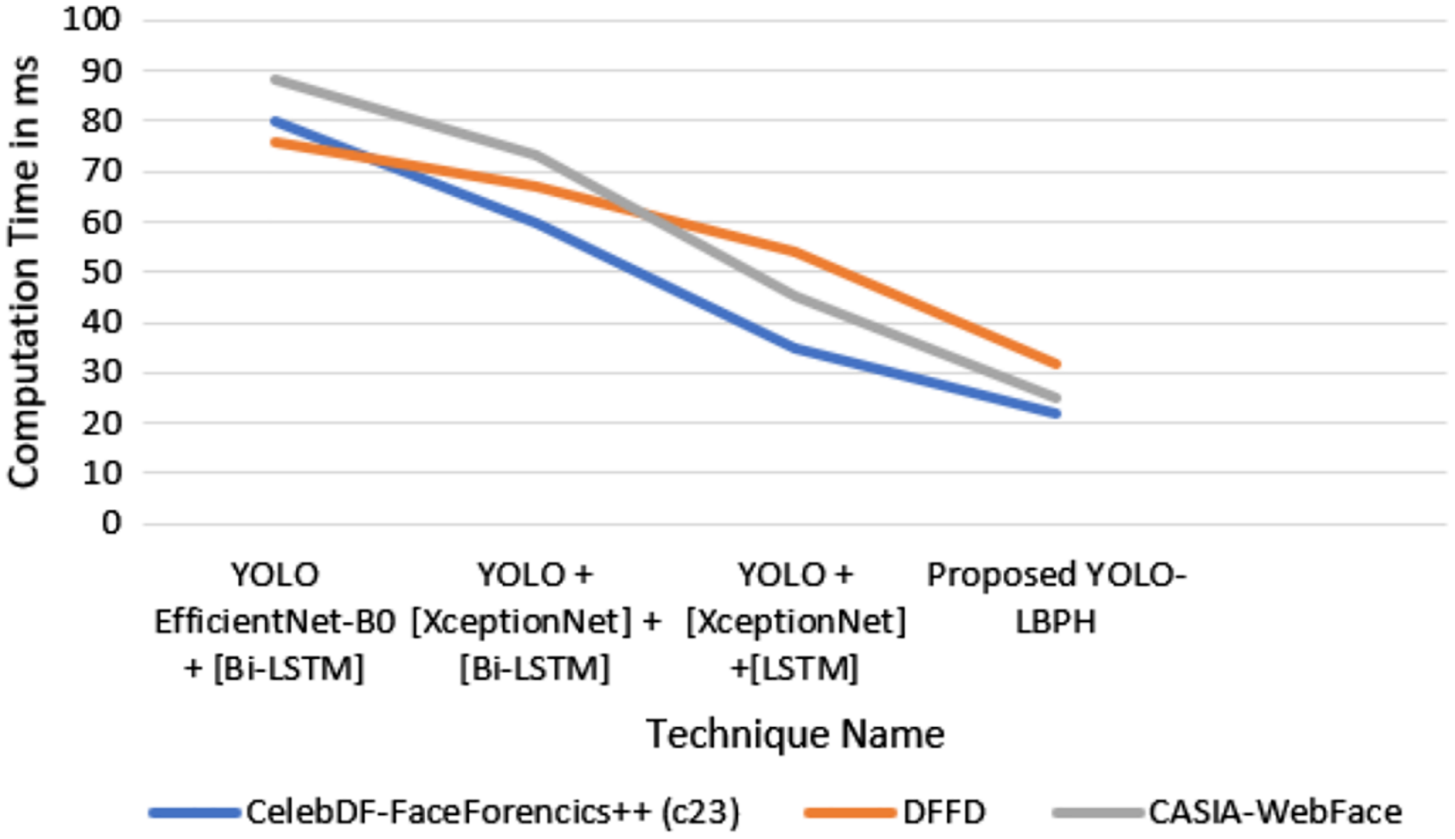
Computation time.

In Fig. 5, it is shown that the proposed algorithm YOLO-LBPH requires less computation time compared to the other existing algorithms. Various frames analyses of same image makes the prediction very fast. The analysis of training and testing of the data set in fake face video images detection and validation of face input data in terms of accuracy and loss metric information is done for the data set of deepfake face images. Figure 6 shows the computation time for various algorithms with various datasets such as Celeb-DF-FaceForencics++(c23), DFFD, CASIA-Web Face dataset.

In Fig. 6, it is shown that the proposed algorithm YOLO-LBPH needs less computation time compared to the other existing algorithms. The analysis of training and testing of the dataset in the detection of fake face video image is presented. The result produces the validation face input data in terms of accuracy and loss metric information for the deepfake face image dataset.

**Figure 7:**
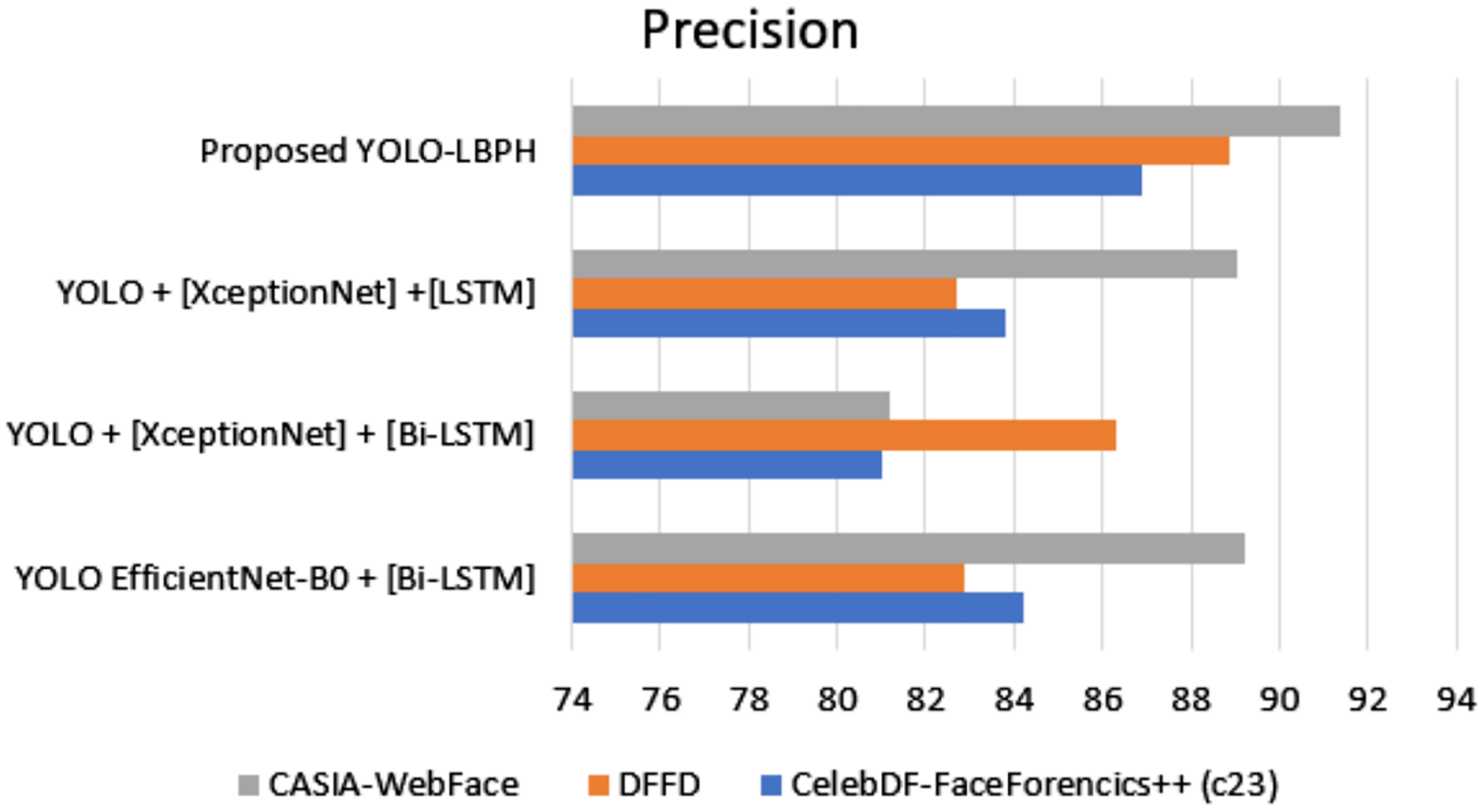
Precision.

Figure 7 shows the precision metric measures of different datasets with different algorithms. The proposed work provides better performance compared to the existing algorithms in various datasets such as Celeb-DF-FaceForencics++(c23), DFFD, and CASIA-Web Face. Here, the proposed YOLO-LBPH has a precision score of 86.88% in the Celeb-DF-FaceForencics++(c23) dataset, 88.9% in the DFFD dataset, and 91.35% in the CASIA-Web Face dataset.

**Figure 8:**
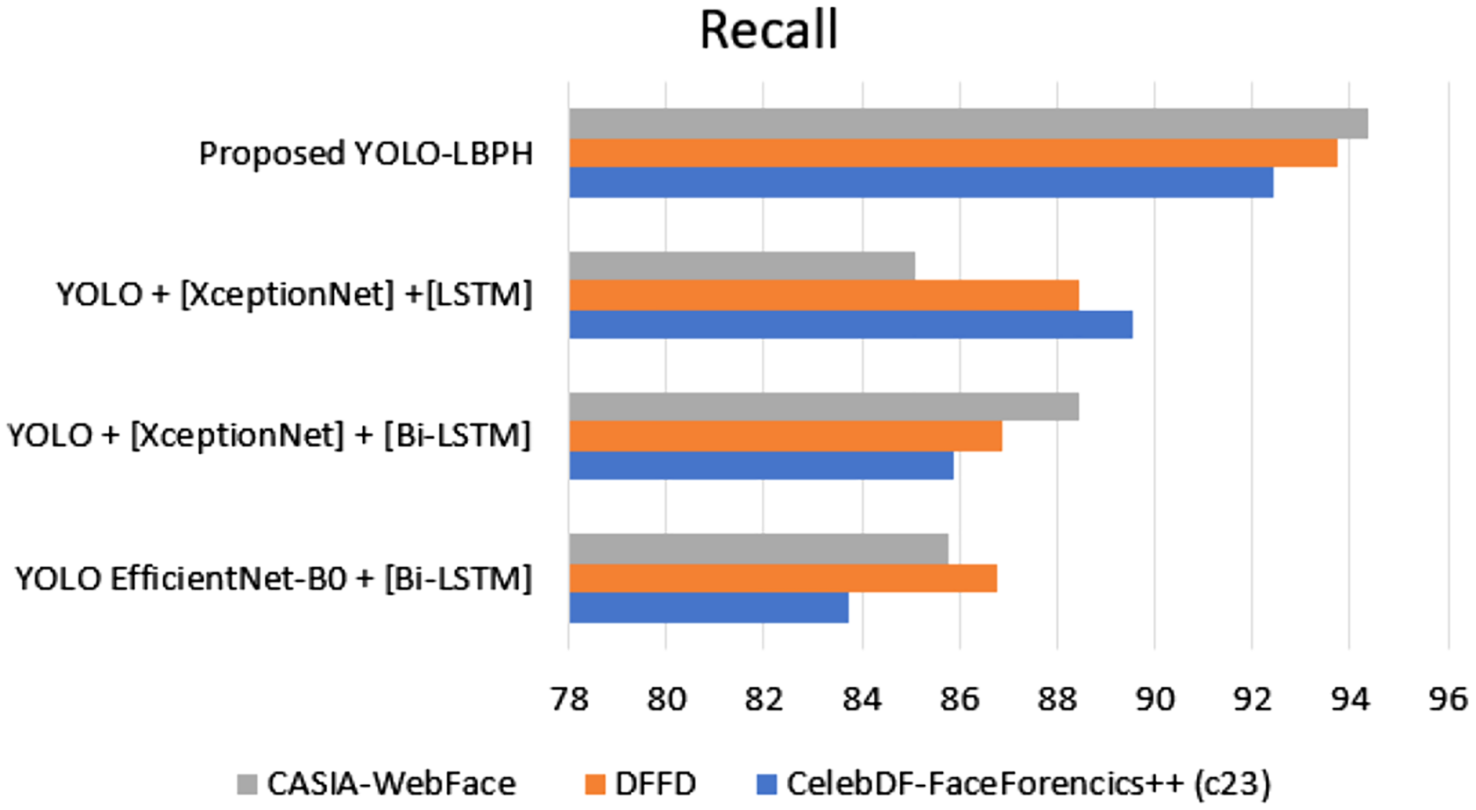
Recall.

Figure 8 shows the recall metric measures of different datasets with different algorithms. The proposed work has provided better performance compared to the existing algorithms. The various datasets of Celeb-DF-FaceForencics++(c23), DFFD, CASIA-Web Face dataset are used in evaluation. We can see that the proposed YOLO-LBPH has a recall score of 92.45% in the Celeb-DF-FaceForencics++(c23) dataset, 93.76% in the DFFD dataset and 94.35% in the CASIA-Web Face dataset.

In Table 3, the F1-Measure of different algorithms was analyzed. The proposed method provides better performance compared to the existing algorithms and various datasets of Celeb-DF-FaceForencics++(c23), DFFD, and CASIA-Web Face. Here, YOLO-LBPH has the F1-measure as 91.45% in the Celeb-DF-FaceForencics++(c23) dataset, 92.88% in the DFFD dataset, and 95.35% in the CASIA-Web Face dataset.

**Table 3:** Performance comparison of the proposed YOLO-LBPH with other YOLO algorithms on different datasets in terms of the F1-Measure.

Methods	Datasets			
	FaceForencies++ (c23)	Celeb-DF	DFFD	CASIA-WebFace
YOLO EfficientNet-B0 + [Bi-LSTM]	82.34	83.23	84.25	81.67
YOLO + [XceptionNet] + [Bi-LSTM]	88.12	84.88	86.56	87.44
YOLO + [XceptionNet] + [LSTM]	88.54	87.56	86.42	83.12
Proposed YOLO-LBPH	91.45	91.92	92.88	95.35

## Conclusion

In this article, You Only Look Once (YOLO) with Linear Binary Pattern Histogram (LBPH) was presented as a face detector for deepfake video detection. This proposed method employs a YOLO-LBPH face detector to detect face regions in video frames. For the extraction of features, EfficientNet-B5 is used. The video frames of the facesv detected at various time slot is compared. The dataset used in this work are Celeb-DF-FaceForencics++(c23), DFFD, CASIA-WebFace. The proposed method achieves high performance deepfake detection scoring in the sense of AUROC, accuracy, recall, precision, and F-measure metrics. AUROC is 88.35% of the score for the Celeb-DF-FaceForencics++(c23). A comparative analysis has revealed that the suggested method outperforms state-of-the-art methods. ArcFace in the YOLO helps to learn the feature from inter and intra circle of respective image. Therefore, the precision score is measured as 86.88% in the Celeb-DF-FaceForencics++(c23) dataset, 88.9% in the DFFD dataset, and 91.35% in the CASIA-WebFace data. Similarly, the recall score is revealed as 92.45% in the Celeb-DF-FaceForencics++(c23) data set, 93.76% in the DFFD dataset, 94.35% in the CASIA-WebFace data set. The limitation of the proposed work is that the images are processed with only local information. Global feature information must be considered in the future. In future research, YOLO-LBPH may be extended to various classifiers and may use different distance metric measures to detect the deepfake face video image.

## Supplemental Information

10.7717/peerj-cs.1086/supp-1 Supplemental Information 1 Implementation.

## Additional Information and Declarations

### Data Availability

The following information was supplied regarding data availability: The data and code is available at GitHub: https://github.com/ondyari/FaceForensics.
